# Transition From Crawling to Walking Changes Gaze Communication Space in Everyday Infant-Parent Interaction

**DOI:** 10.3389/fpsyg.2019.02987

**Published:** 2020-01-24

**Authors:** Hiroki Yamamoto, Atsushi Sato, Shoji Itakura

**Affiliations:** ^1^Graduate School of Letters, Kyoto University, Kyoto, Japan; ^2^Faculty of Human Development, University of Toyama, Toyama, Japan; ^3^Center for Baby Science, Doshisha University, Kizugawa, Japan

**Keywords:** social interaction, interpersonal distance, eye contact, crawling, walking, second-person perspective, head-mounted eye tracking

## Abstract

Acquisition of walking changes not only infants' locomotion itself but also infants' exploratory behavior and social interaction, such as gaze communication. To understand the ecological context in which gaze communication occurs and how it changes with walking development from the point of view of the spatial arrangement of infants, parents, and objects, we analyzed longitudinal data of daily eye contact scenes recorded from head-mounted eye trackers worn by parents as infants grew from 10 to 15.5 months, focusing on infant-parent distance and the number of objects between the dyad. A Bayesian state-space model revealed that the interpersonal distance at which infants initiated eye contact with their parents increased with the time ratio of walking to crawling. This result could not be explained by the developmental change in the amount of time that the infants were far from the parents, which is not limited to the gaze communication context. Moreover, the interpersonal distance at which the parents initiated eye contact with the infants did not increase with the time ratio of walking to crawling. The number of objects on the floor between infants and parents at the time of eye contact increased with interpersonal distance. Taken together, these results indicate that the transition from crawling to walking changes the ecological context in which infants initiate gaze communication to a visual environment characterized by a larger interpersonal distance and, therefore, more objects cluttered between the dyad. The present study has wider implications for the developmental change of shared attention in conjunction with walking development.

## 1. Introduction

One aim of developmental science is to explain phenomena that occur in infants' everyday lives. Developmental theories often make hypotheses, assumptions, and implications, known as “ecological commitments,” about what happens in infants' daily lives outside research contexts (Dahl, 2017). To test or support ecological commitments empirically, it is important to investigate infants' lived experiences in naturalistic environments as well as randomized control tests in experimental rooms. Recent advances in technology and analytical methods have made it possible to evaluate what and how infants see around them or hear in their everyday experience, such as their daily language environment (Roy et al., 2015; Bergelson et al., 2019) and visual experience of faces (Jayaraman et al., 2015; Jayaraman and Smith, 2019), hands (Fausey et al., 2016) or objects (Clerkin et al., 2017; Suanda et al., 2019).

Acquisition of walking is one factor that changes how infants see the world in everyday lives because our first-person perspective visual experiences are shaped by our bodies. Compared with crawling infants, walking infants have higher and more distant visual fields (Kretch et al., 2014). The development of wearable eye trackers for free-moving infants has demonstrated that acquisition of walking changes not only infants' visual exploration but also gaze communication between infant and parent. While moving on a walkway in the laboratory room, walking infants directed their gaze straight ahead at parents in front of them, whereas crawling infants looked down at the floor (Kretch et al., 2014). A similar tendency was confirmed in the situation where both infant and parent could move freely in the laboratory room. Infants with an upright or sitting posture were more likely to look at parent's faces and engage in eye contacts than infants with prone postures (Franchak et al., 2018). These studies suggest that the change in infants' first-person perspective accompanied by a change in their locomotion or posture affects the frequency of infants' social looks and eye contact.

The question that remains to be answered is how the situation in which gaze communication occurs in daily lives changes along walking development. Unlike gaze behavior directed toward a social stimulus presented on the monitor in the experimental room, daily gaze communication in the real world is embedded in the three-dimensional space. In the space, both infants and parents can move around freely, and many objects are arranged in a complex manner. In such a messy environment, the transition from crawling to walking changes not only infants' locomotion itself, but also infants' interaction with objects and people. Compared to crawlers, walkers move more, see more, play more, and interact more (for reviews, see Adolph and Tamis-LeMonda, 2014). After the onset of walking, infants take more steps, travel farther distances, and fall less (Adolph et al., 2012). The elevated vantage point of walkers enables them to see distant objects (Kretch et al., 2014), and the hands that become free from supportive functions allow access to and carrying of distant objects (Karasik et al., 2011, 2012; Dosso and Boudreau, 2014). Moreover, walkers are more likely to approach their parents to share objects (Karasik et al., 2011) and make vocalizations and gestures directed to their parents (Clearfield, 2011). Taken together, it is no wonder that the ecological context in which gaze communication occurs may also change as infants' interaction with objects and people changes with walking development.

The aim of the current study is to investigate how the ecological context in which daily gaze communication occurs changes with walking development from the point of view of the spatial arrangement of the infant, the parents, and objects. The interpersonal distance and relative arrangement of objects influence infant-parent gaze communication by interacting with the magnitude of the gesture and the infant's age (Butterworth and Jarrett, 1991; Deák et al., 2000; Flom et al., 2004; Gonseth et al., 2013; Yamamoto et al., 2019). The infant-parent distance modulates the smooth exchange of eye contact and influences the infant's and the parent's social looks differently (Yamamoto et al., 2019). In referential gaze communication, distractor objects in the visual environment often disturb young infants' detection of what their parents refer to, and noticeable gestures from parents are needed to coordinate their visual attention (Butterworth and Jarrett, 1991; Flom et al., 2004). Although the acquisition of walking changes the interaction between infants and objects or people, little is known about the developmental change of such spatial arrangements as that of infants, parents, and objects. In general, gaze communication is the basis of social learning in infants, and it leads to later language development and theory of mind (Brooks and Meltzoff, 2005, 2015). Thus, to understand how the acquisition of walking shapes new opportunities for social learning, it is important to describe the developmental changes in the daily visual environment in which gaze communication occurs between the infant and the parent, that is, the interpersonal distance and the degree of object clutter at the time of gaze communication.

Despite the importance of describing daily gaze communication, few studies have investigated the gaze communication between a free-moving dyad's in everyday life. This is probably due to several methodological considerations. When recording from a third-person perspective, accurate scoring of an infant's gaze behavior is dependent on the complexity of the environment and position of the video camera (Franchak, 2020a), making this method unsuitable for measuring a free-moving dyad's gaze communication. In fact, many previous studies that measured daily gaze communication set infant and parent at a fixed interpersonal distance so that the participants remained visible in the video cameras (e.g., Deák et al., 2014; de Barbaro et al., 2016). One alternative method is recording from the infant's first-person perspective with a wearable eye tracker. Several studies used this method to measure free-moving infants' visual exploration in a laboratory room. However, in previous studies, an experimenter had to walk with infants to prevent infant injury from face-first falls (e.g., Kretch et al., 2014; Franchak et al., 2018; Hoch et al., 2019b). Thus, this method is also not suitable for the purpose of recording daily gaze communication.

One solution to investigating a free-moving dyad's gaze communication in everyday life is recording face-to-face interaction from the parent's first-person perspective, that is, the infant's second-person perspective. A head-mounted camera worn by the infant's social partner allows for measuring eye contact during a live social interaction more reliably and more validly than when using a stationary camera (Edmunds et al., 2017). Moreover, by using this method, we previously recorded a free-moving dyad's daily eye contact scenes longitudinally and demonstrated that interpersonal distance affects the infant's and parent's social looks differently (Yamamoto et al., 2019).

In this study, taking advantage of the recording from the infant's second-person perspective as above, we show how the transition from crawling to walking changes the ecological context in which gaze communication occurs in everyday life. This study is an extension of a previous report (Yamamoto et al., 2019) using the same longitudinal dataset. We investigated the developmental change in the spatial arrangement of infants, parents, and objects where gaze communication occurred, focusing on (i) infant-parent distance and (ii) the number of objects between them at the time of eye contact. Regarding the interpersonal distance at the time of eye contact, we predict that the effect of walking development would vary depending on who initiates eye contact with the social partner. After the onset of walking, the elevated vantage point leads infants to see and access distant objects or people (Karasik et al., 2011; Kretch et al., 2014), but there is no such change for parents. If an infant's visual exploration leading to gaze communication is shaped by the infant's first-person perspective view, the interpersonal distance at the time of eye contact would increase with the infant's walking development only for the eye contact the infant, not the parent, initiates. Regarding the number of objects at the time of eye contact, a previous study has showed that walkers are more likely to carry objects and approach their parents to share these objects than are crawlers (Karasik et al., 2011). Thus, we predict that the visual environment between infant and parent will become cluttered with more objects consistent with the infant's walking development. We evaluate the effect of walking development on the number of objects between the dyad while controlling for the effect of interpersonal distance because the number of objects on the floor between the dyad is expected to increase with the interpersonal distance irrespective of the infant's walking development.

## 2. Materials and Methods

### 2.1. Participants and Data Collecting

Five healthy, full-term infants (1 male and 4 female; A–E) and their mothers contributed to the present study, beginning when the infant was 10 months old and ending when the infant was 15.5 months of age. All participants were of Japanese ethnicity. This sample was taken from a longitudinal study investigating the effect of interpersonal distance on infant-parent gaze communication by Yamamoto et al. (2019).

**Figure 1:**
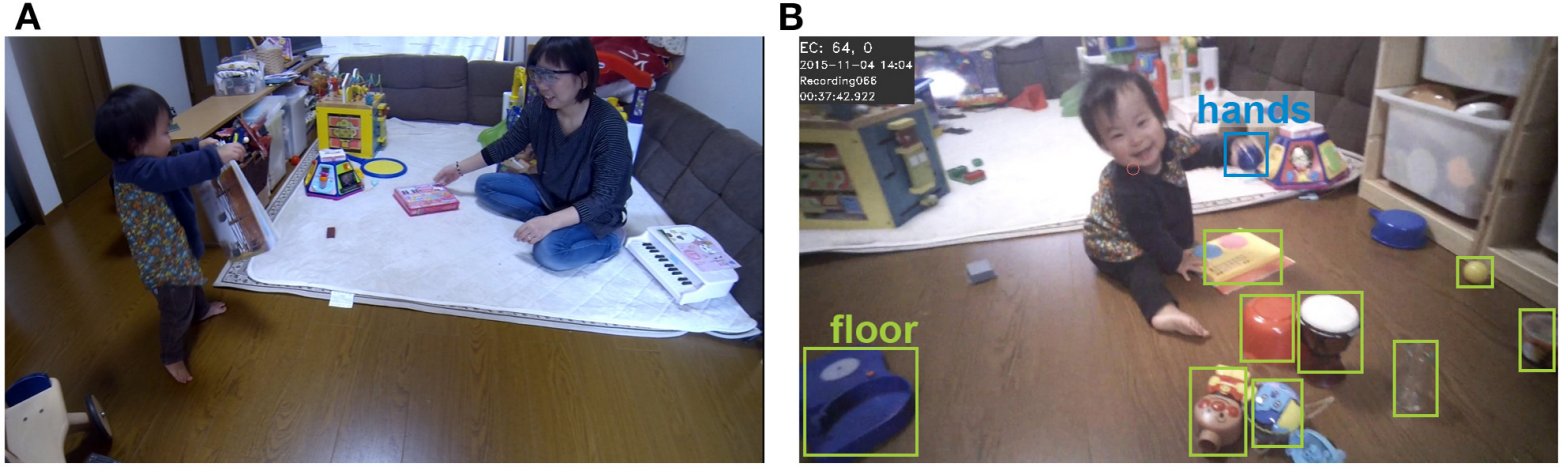
**(A)** We observed daily infant-parent interaction using head-mounted eye trackers worn by the parents. The infants and parents could move freely in their home environment. **(B)** Image at the time of eye contact captured from the scene camera of a head-mounted eye tracker worn by a parent. For each eye contact scene, we counted the number of objects on the floor between the dyad (light green) and objects in infant's hands (blue).

We visited each participant's home on alternate weeks and recorded infants' and parents' daily activities from a head-mounted eye tracker (Tobii Glasses 2, Tobii Technology) worn by parents for up to 1.5 h each day. Before every recording, the parent wearing the head-mounted eye tracker was instructed to look at and focus on the center of a card with a black-and-white target held at arm's length, and a calibration was then performed using eye-tracking software (Tobii Glasses Controller). We told parents that we were just interested in the infants' everyday activity and infants and parents could engage in any daily activities, go anywhere in their home and play with any of the available toys (see Figure 1A). After the observation, we measured the infant's face size (between the chin and the eyebrows).

For each observation day, we also recorded infant-parent social interaction with a head-mounted camera (GoPro Hero4, Woodman Labs) worn by H.Y. so that participants could be seen on the head-mounted camera as much as possible. However, due to a malfunction of the battery, we were unable to record videos from the head-mounted camera on several observation days (when infant A was 12.5 months of age, when infant D was 14.5 months of age, and when infant E was 15.5 months of age).

We could not collect data when infant E was 12 months of age because infant E was in poor physical health. The mean observation time for each day was 1 h 25 min, and the mean total observation time for one infant was 16 h 48 min. Extensive details regarding data collection and the age in days for each observation day are included in the report by Yamamoto et al. (2019).

All infants participated with written informed consent from their parents. In addition, for publication of identifying images in an online open-access publication, we obtained informed consent from the parents of the infant, as shown in Figure 1. This research was approved by the ethics review board at the Unit for Advanced Studies of the Human Mind, Kyoto University (27-P-6) and was conducted in accordance with the Helsinki Declaration guidelines and regulations.

### 2.2. Data Processing

Using video recordings from the parent's point of view, we performed five data processing steps (Yamamoto et al., 2019). (i) We coded 3 types of infant locomotion—“crawling,” “cruising,” and “walking”—with one-zero sampling (Altmann, 1974) for 15 s and calculated the proportion of the infant's walking time to the sum of walking time and crawling time for each observation day. (ii) By checking the parent's perspective video frame by frame, we identified the video frame of each eye contact. The video frame of the eye contact was defined as the video frame of the “parent looking at the infant's face,” and the infant's gaze was coded as “directed to the parent” by the coders. Coding whether the infant's gaze was directed to the parent from the parent's perspective video was based on Edmunds et al. (2017). We defined continuous eye contact video frames that included glances from either partner for less than 1 s but no longer than 1 s as an eye contact bout (EC bout). (iii) By checking which partner initiated the eye contact, we categorized an EC bout as either an infant-led or parent-led EC bout. (iv) We defined an eye contact session (EC session) as a series of EC bouts with short inter-EC-bout intervals and used it as an independent observation unit because EC bouts usually occurred intermittently. (v) The monocular camera generates a one-to-one relationship between the object and the image. Using the video frames of EC bouts, we estimated the interpersonal horizontal distance at the time of the EC bouts from the accelerometer data from the head unit, the focal length, and the real and pixel size of the infant's face (between the chin and the eyebrows). Extensive details regarding data processing were included in the report by Yamamoto et al. (2019).

The aim of the present study was to investigate developmental changes in the spatial arrangement of infants, parents, and objects where daily gaze communication occurs, focusing on infant-parent distance and the number of objects between them. With this aim, we newly coded two measures: the proportion of distance category and the number of objects between the dyad.

#### 2.2.1. Proportion of Distance Category

To understand the relation between walking development and the infant-parent distance at which gaze communication occurs, it is also necessary to investigate the usual infant-parent distance, which is not limited to the gaze communication context. If an infant's walking development increases the time the infant is far from their parents at various daily contexts, and if gaze communication occurs randomly and irrespective of context, then it is no wonder that the interpersonal distance at the time of eye contact increased with walking development. However, if the developmental change of an infant's first-person perspective shapes the interpersonal distance in face-to-face interactions, then an increase in the interpersonal distance consistent with walking development might occur more clearly in the gaze communication context than in other contexts. To investigate whether the developmental change of the interpersonal distance at the time of eye contact could be simply explained by the developmental change of the usual interpersonal distance, we need to evaluate the usual interpersonal distance for each observation day.

We coded infant-parent distance using the recording from a head-mounted camera worn by H.Y. We coded infant-parent distance into four ordered categories, “0–0.5 m,” “0.5–1.0 m,” “1.0–1.5 m,” and “1.5 m or more,” with instantaneous sampling (Altmann, 1974) for every 30 s. There were some instantaneous samples in which the infants' movement had been constrained by the parent or environmental objects. For example, infants were sometimes put in playpens when the parent did not want to be disrupted by the infants in order to do light housekeeping. Moreover, infants were sometimes held or carried by their parents in social interactions. We did not code such instantaneous samples because infants could not adjust the interpersonal distance. We calculated the proportion of each distance category for each observation day. The second coder independently judged a randomly selected 20% of the video, with 75% intercoder agreement (*kappa* = 0.77).

#### 2.2.2. Number of Objects Between Infant and Parent

Using the head-mounted eye-tracker worn by the parents, we output the first frame of each EC bout and coded the number of objects placed between infant and parent. We counted the number of objects that infants could lift from the floor, such as balls or toys and not tables or sofas. This definition was based on the concepts of “detached objects” and “attached objects” from Gibson (1979). Using this definition, we prioritized foreground, not background, objects (the book on the floor and not the floor itself).

Sometimes, there were EC bouts in which it was difficult to accurately count individual objects, such as balls in a basket. In such situations, multiple objects were nested within another object, and one object could be covered with another object. Because it was difficult to count the exact number of nested objects at the micro level, we counted the macroscopic visual unit as one object. In case of “balls in a basket,” we counted all the balls and the basket as one object.

Some objects were sometimes held by the infant or parent. For each EC bout, we counted the number of objects between infant and parent in each of object locations, “on the floor” or “in infant's hands” (see Figure 1B). The second coder independently judged a randomly selected 20% of the EC bouts with average 81.6% intercoder agreement (on the floor: 78%; in infant's hands: 85%), and the numbers of objects were correlated (on the floor: *r* = 0.55; in infant's hands: *r* = 0.80). We removed three EC bouts (0.001% of total EC bouts) because of a difficulty in coding from an image blur, and finally, we analyzed 3135 EC bouts.

### 2.3. Data Analysis

We conducted three main statistical analyses using Bayesian state-space models. The core of the state-space model is a generalized linear mixed model (GLMM) (Baayen, 2008) used to estimate the effects of various explanatory variables on the response variables measured from longitudinal observation data and considering the effects of temporal autocorrelation. Analysis 1 was intended to estimate the effect of walking development on the infant-parent distance at which eye contact occurs. Analysis 2 was intended to estimate the effect of walking development on the usual infant-parent distance, which is not limited to the gaze communication context. Analysis 3 was intended to estimate the effect of walking development on the number of objects between infant and parent. In each analysis, we estimated the coefficient parameters of the explanatory variables. If the parameter estimate of one explanatory variable is positive, it can be interpreted that the response variable increases with the value of the explanatory variable, while controlling for the effects of the other explanatory variables. If the 95% credible interval of the parameter does not include zero, it can be inferred that there is a significant effect, as seen in classic statistical hypothesis testing.

In a previous study, we found that infant-led EC occurs at a greater interpersonal distance than parent-led EC (Yamamoto et al., 2019). The purpose of Yamamoto et al. (2019) was to evaluate the effect of infant-parent distance on daily gaze communication between the dyad, and the response variable was the number of EC bouts within the EC session. Contrary to Yamamoto et al. (2019), the purpose of the current study is to evaluate the effects of an infant's walking development on infant-parent distance (Analysis 1) or the number of objects between the dyad (Analysis 3) at the time of eye contact. Although the purpose and response variables were different from those in the previous study, we used the same sample in this study as that used in the previous study. To avoid redetecting the previously reported effects involving the initiator of eye contact, we divided the data into two subsets by the initiator of the EC bouts, and we individually analyzed infant-led EC bouts and parent-led EC bouts in Analysis 1 and Analysis 3, respectively.

In Analysis 1, the response variable was the infant-parent distance of an infant-led EC bout or parent-led EC bout following a lognormal distribution. The explanatory variables were infant age and proportion of infant's walking time for each observation day. Analysis 2 was a kind of mixed ordered logistic regression controlling for the effects of temporal autocorrelation, and the response variable was the proportion of each distance category for each observation day. The explanatory variables were the same as in Analysis 1. In Analysis 3, the response variable was the number of objects on the floor between the dyad or objects in the infant's hands at the time of an infant-led EC bout or parent-led EC bout following a Poisson distribution. The explanatory variables were infant age, proportion of infant's walking time for each observation day, and infant-parent distance at each EC bout. To consider differences in the EC session, we set the EC session as a random intercept in Analysis 1. In Analysis 3, we set the EC session as a random intercept for only the analysis of objects on the floor because this setting made the Markov chains convergence difficult for the analysis of objects in the infant's hands. In all analyses, we chose weakly informative priors for the hyperprior of system noise because they helped to stabilize parameter estimates (Gelman et al., 2013). More details on the statistical models are described in the Supplementary Material.

All models were fitted using the Hamiltonian Monte Carlo engine Stan 2.19.2 (Stan Development Team, 2019) in R 3.6.0 (R Core Team, 2019). All iterations were set to 11,000, and burn-in samples were set to 1000 with the number of chains set to four. The values of Rhat for all parameters were below 1.1, indicating convergence across the four chains (Gelman et al., 2013). To check our approach, we simulated the hypothesized data-generating process using the posterior median 50 times, and we iteratively estimated each model in Analysis 1 and Analysis 3.

## 3. Results

Although there were individual differences in motor development, all infants changed their locomotion in daily use from crawling to walking over the longitudinal observation (see Figure S1).

### 3.1. Infant-Parent Distance at Which Eye Contact Occurs

Referring to the 95% credible interval (CI) of the posterior distributions of the fixed effect parameters (Table 1), the proportion of the infant's walking time had a clearly detected effect on the infant-parent distance at which an infant-led EC bout occurs (mean = 0.005, 95% CI = [0.0002, 0.009]) because the 95% CI did not include zero. Contrary to an infant-led EC bout, the proportion of the infant's walking time did not have a clearly detected effect on infant-parent distance at which a parent-led EC bout occurs (mean = 0.0007, 95% CI = [−0.002, 0.004]). The 95% CIs of the effect of age on infant-parent distance included zero for both infant-led EC bouts (mean = −0.053, 95% CI = [−0.119, 0.010]) and parent-led EC bouts (mean = −0.010, 95% CI = [−0.053, 0.032]).

**Table 1:** The posterior distribution of the parameters of the model.

**Analysis**	**Response variable**	**Explanatory variable**	**EAP**	**2.5%**	**97.5%**
Analysis 1	Distance (Infant-led EC)	Age	−0.053	−0.119	0.010
		Walking time	0.005	0.0002	0.009
	Distance (Parent-led EC)	Age	−0.010	−0.053	0.032
		Walking time	0.0007	−0.002	0.004
Analysis 2	Proportion of distance category	Age	0.016	−0.131	0.193
		Walking time	0.005	−0.006	0.014
Analysis 3	Number of objects on the floor	Age	−0.041	−0.180	0.092
	(Infant-led EC)	Walking time	−0.002	−0.013	0.009
		Distance	0.944	0.750	1.15
	Number of objects on the floor	Age	0.016	−0.135	0.159
	(Parent-led EC)	Walking time	−0.012	−0.024	0.00002
		Distance	1.63	1.26	2.00
	Number of objects in infant's hands	Age	−0.032	−0.096	0.028
	(Infant-led EC)	Walking time	0.003	−0.002	0.008
		Distance	−0.051	−0.176	0.068
	Number of objects in infant's hands	Age	−0.024	−0.096	0.042
	(Parent-led EC)	Walking time	0.001	−0.004	0.006
		Distance	0.144	−0.035	0.312

*The mean (EAP) and quantiles (2.5% and 97.5%) of the posterior distribution are shown*.

**Figure 2:**
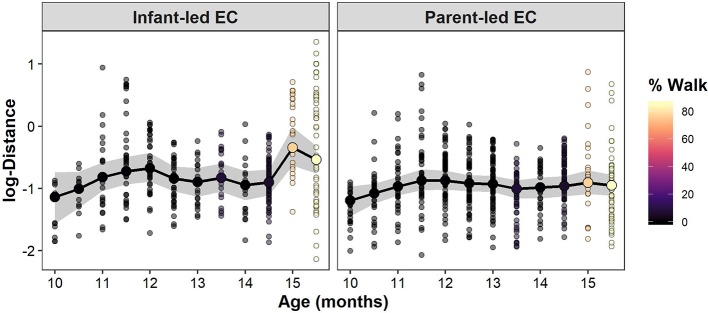
Longitudinal development of interpersonal distance at which infant-led EC bouts **(left)** occur and interpersonal distance at which parent-led EC bouts **(right)** occur in one representative infant (infant A). The posterior mean (large colored dots) and 95% credible interval (gray areas) of the mean interpersonal distance of EC bouts in each observation day are shown. The observed data are represented with small colored dots. The color of the dots represents the proportion of the infant's walking time to the sum of walking time and crawling time for each observation day. Note that the interpersonal distance of each EC bout (meters) is log transformed.

**Figure 3:**
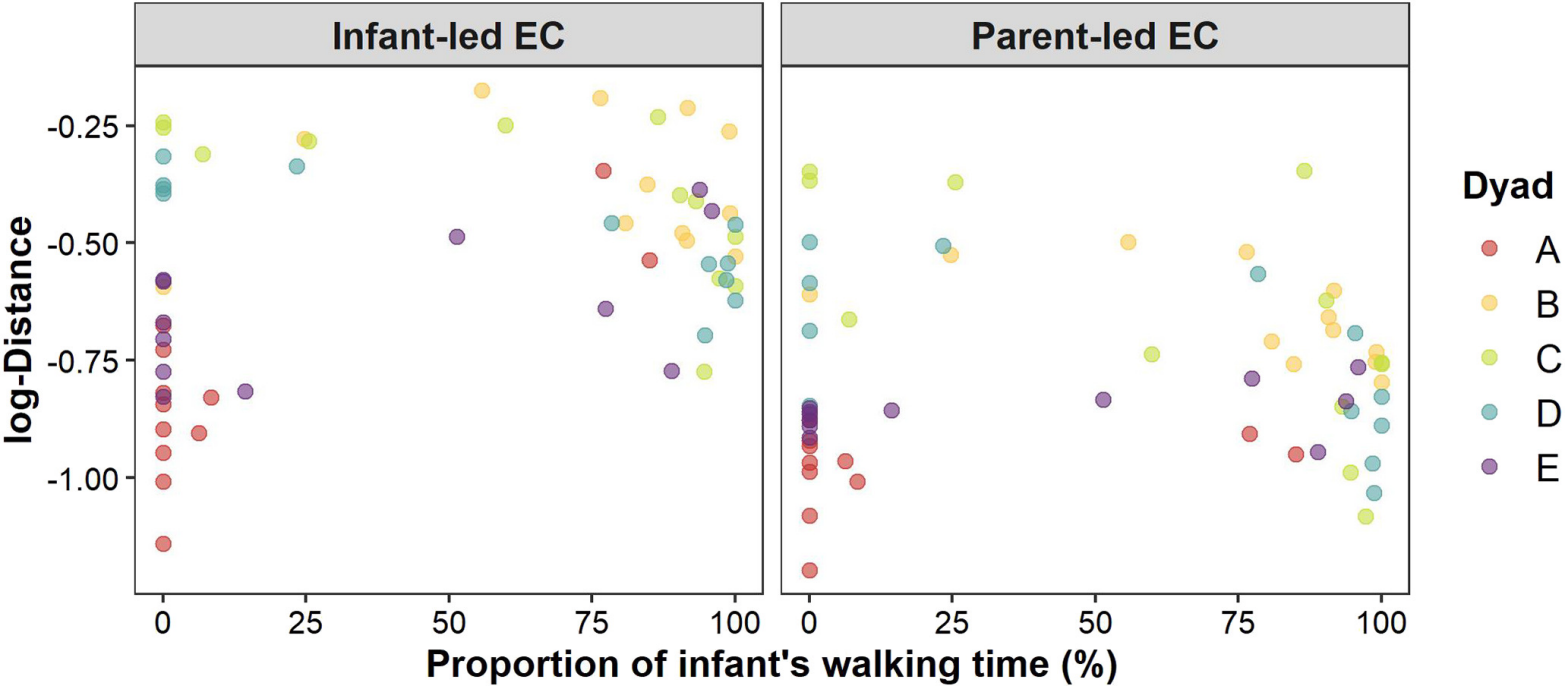
Relationship between the proportion of infant's walking time and the posterior mean of the interpersonal distance (log transformed) at the time of infant-led EC bouts **(left)** and parent-led EC bouts **(right)**. Each dot represents one observation day. The color of the dots represents each infant-parent dyad.

Figure 2 shows the longitudinal development of the infant-parent distance at which eye contact occurs for a representative infant-parent dyad (see Figure S2). Because the effect of walking time had a positive value only for infant-led EC bouts, the predictions of the infant-parent distance at the time of eye contact increased with the proportion of the infant's walking time only for infant-led EC bouts but not for parent-led EC bouts. We can also confirm this tendency from predictions of the mean infant-parent distance at the time of eye contacts for each observation day from all infant-parent dyads (Figure 3).

To check our approach, we simulated new time series data and estimated the parameters of the model with the new data 50 times. Regarding the infant-led EC bouts, the Markov chains converged 49 times, and the 95% CIs of the posterior distributions of the fixed effect parameters did not include zero 18 times (36.7%) for the effect of age, and they did not include zero 29 times (59.2%) for the effect of walking time. Regarding the parent-led EC bouts, the Markov chains converged 50 times, the 95% CIs of the posterior distributions of the fixed effect parameters did not include zero 5 times (10%) for the effect of age, and they did not include zero 8 times (16%) for the effect of walking time.

### 3.2. Proportion of Distance Category

Referring to the 95% CI of the posterior distributions of the fixed effect parameters (Table 1), both the proportion of infant's walking time (mean = 0.005, 95% CI = [−0.006, 0.014]) and age (mean = 0.016, 95% CI = [−0.131, 0.193]) had no clearly detected effects on the proportion of distance category because the 95% CIs included zero. This result suggests that the usual infant-parent distance that is not limited to the gaze communication context did not change with the proportion of infant's walking time when controlling for the effect of temporal autocorrelation and infant age (see Figure S3).

### 3.3. Number of Objects Between Infant and Parent

Regarding the objects on the floor, referring to the 95% CIs of the posterior distributions of the fixed effect parameters (Table 1), the 95% CIs of the effect of walking time on the number of objects between the dyad included zero for both infant-led EC bouts (mean = −0.002, 95% CI = [−0.013, 0.009]) and parent-led EC bouts (mean = −0.012, 95% CI = [−0.024, 0.00002]). The 95% CIs of the effect of age also included zero for both infant-led EC bouts (mean = −0.041, 95% CI = [−0.180, 0.092]) and parent-led EC bouts (mean = 0.016, 95% CI = [−0.135, 0.159]). Infant-parent distance had a clearly detected effect on the number of objects between the dyad for both infant-led EC bouts (mean = 0.944, 95% CI = [0.750, 1.15]) and parent-led EC bouts (mean = 1.63, 95% CI = [1.26, 2.00]) because the 95% CIs did not include zero.

Regarding the objects in the infant's hands, the 95% CIs of all effects on the number of objects included zero for both infant-led EC bouts (age: mean = −0.032, 95% CI = [−0.096, 0.028]; walking time: mean = 0.003, 95% CI = [−0.002, 0.008]; distance: mean = −0.051, 95% CI = [−0.176, 0.068]) and parent-led EC bouts (age: mean = −0.024, 95% CI = [−0.096, 0.042]; walking time: mean = 0.001, 95% CI = [−0.004, 0.006]; distance: mean = 0.144, 95% CI = [−0.035, 0.312]).

**Figure 4:**
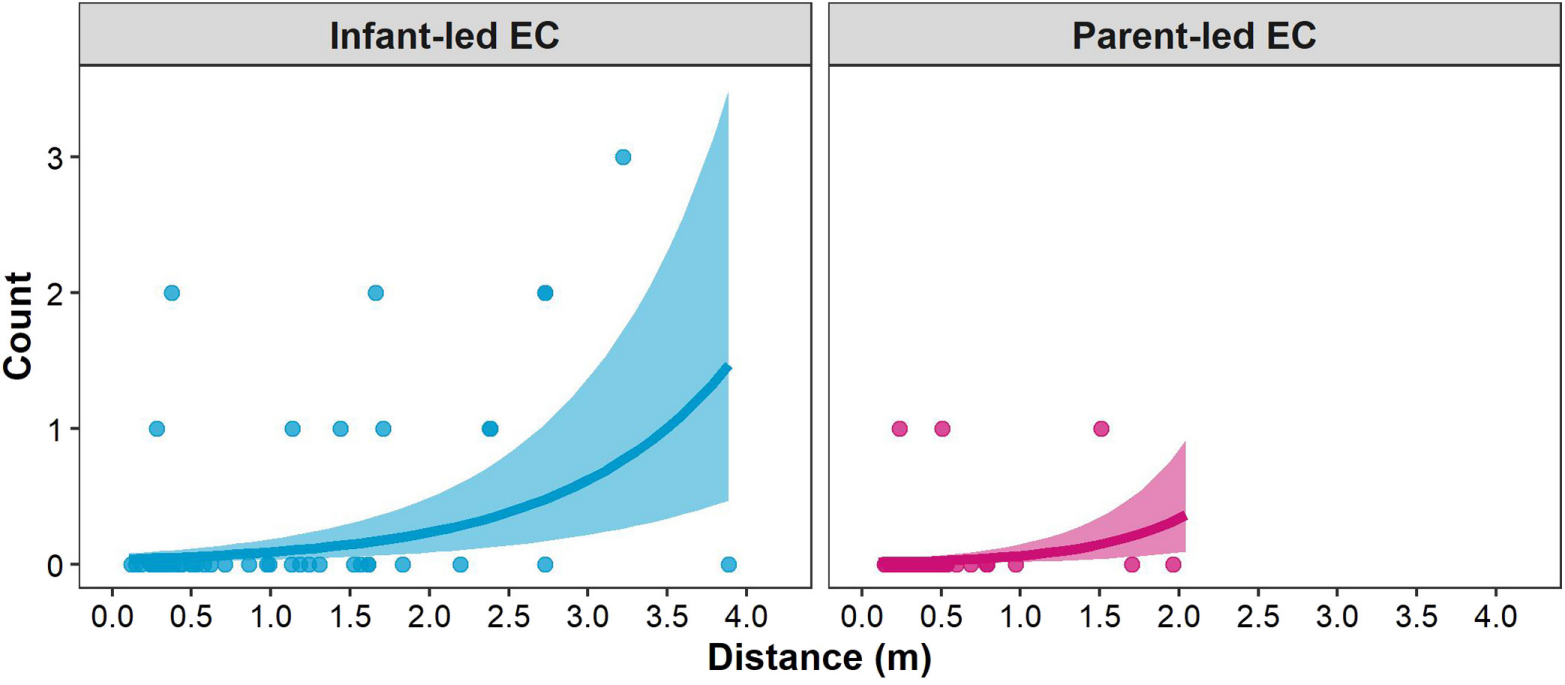
Relationship between the infant-parent distance and number of objects on the floor between the dyad for infant-led EC bouts (left panel; cyan) and parent-led EC bouts (right panel; magenta) on one observation day of one infant (when infant A was 15.5 months of age). The posterior mean (colored lines) and 95% credible interval (colored areas) of the mean number of objects between the dyad are shown. The colored dots represent the observed data.

Figure 4 shows the predictions for the number of objects on the floor between a dyad for a representative observation day of one infant-parent dyad (see Figures S4, S5). Because the effect of interpersonal distance had a positive value for both infant-led EC bouts and parent-led EC bouts, the predictions of the number of objects on the floor between the dyad increased with interpersonal distance regardless of whether the eye contact was initiated by the infant or the parent.

To check our approach, we simulated new time series data and estimated the parameters of the model with new data 50 times. Regarding the number of objects on the floor at the time of infant-led EC bouts, the Markov chains converged 48 times, and the 95% CIs of the posterior distributions of the fixed effect parameters did not include zero 0 times (0%) for the effect of age, they did not include zero 2 times (4.2%) for the effect of walking time, and they did not include zero 48 times (100%) for the effect of interpersonal distance. Regarding the number of objects on the floor at the time of parent-led EC, the Markov chains converged 48 times, and the 95% CIs of the posterior distributions of the fixed effect parameters did not include zero 3 times (6.3%) for the effect of age, they did not include zero 37 times (77.1%) for the effect of walking time, and they did not include zero 48 times (100%) for the effect of interpersonal distance. Regarding the number of objects in the infant's hands at the time of infant-led EC bouts, the Markov chains converged 48 times, and the 95% CIs of the posterior distributions of the fixed effect parameters did not include zero 4 times (8.3%) for the effect of age, they did not include zero 8 times (16.7%) for the effect of walking time, and they did not include zero 5 times (10.4%) for the effect of interpersonal distance. Regarding the number of objects in the infant's hands at the time of parent-led EC, the Markov chains converged 47 times, and the 95% CIs of the posterior distributions of the fixed effect parameters did not include zero 4 times (8.5%) for the effect of age, they did not include zero 4 times (8.5%) for the effect of walking time, and they did not include zero 17 times (36.2%) for the effect of interpersonal distance.

## 4. Discussion

This is the first study to describe the ecological contexts in which gaze communication occurs and how it changes with walking development in infant's everyday lives. With eye contact scenes recorded from infant's second-person perspective, we evaluated the longitudinal change in the spatial arrangement of infants, parents, and objects at the timing of eye contacts, focusing on infant-parent distance (Analysis 1 & Analysis 2) and the number of objects between them (Analysis 3).

### 4.1. Infant-Parent Distance at Which Eye Contact Occurs

In Analysis 1, interpersonal distance for infant-led EC bouts increased along with the proportion of infant's walking time. This result suggests that the interpersonal distance at which gaze communication occurs from infants increases with walking development. This result could not be explained by mere developmental change to the usual infant-parent distance, which is not limited to the gaze communication context. In Analysis 2, the proportion of distance category was not associated with the proportion of infant's walking time, suggesting that the amount of time that infants are farther away from their parents did not change with walking development. Moreover, interpersonal distance for parent-led EC bouts did not show a clear change with the proportion of infant's walking time in Analysis 1. These results suggest that the transition from crawling to walking increases the interpersonal distance at which gaze communication is initiated only for infants and not for parents.

Although previous studies have reported that infant-parent distance or time away from parents increased with motor development or age (Jayaraman et al., 2015; Thurman and Corbetta, 2017, 2019; Hoch et al., 2019a; Jayaraman and Smith, 2019), we could not observe an increase in time away from the parent with walking development or age. There are several possible reasons for the discrepancy between this study and previous studies. First, the size of participants' houses may have limited the possible range of infant-parent distance. Motor development is shaped by social and cultural factors because infants grow up in everyday environments (Adolph and Hoch, 2019). Generally, houses are smaller in Japan than in other countries, and such a cultural difference may lead to no change in infant-parent distance in this study. Second, coarse coding with ordinal scales may have prevented detection of the effects found in previous studies. Third, the small sample size may have made it difficult to detect the effect of infant's walking or age. Depending on the situation for data collection or behavioral measures, it is possible that the daily positioning of the infant and parent has expanded with infant age or walking development. However, using the measure of interpersonal distance at the timing of eye contact, the interpersonal distance at which infants initiated eye contact increased with walking development, while the interpersonal distance at which parents initiated eye contact did not change. Such differences in the development of interpersonal distance at the timing of eye contact cannot be explained simply by the usual interpersonal distance, even if the daily positioning of the dyad expanded with walking development. Taken together, these results suggest that at least the space at which gaze communication is initiated by the infant, not by the parent, expands with infants' walking development.

The current study adds to a growing body of research demonstrating that infants' visual experiences are tied to their posture (Kretch et al., 2014; Franchak et al., 2018). Walking infants have higher and more distant visual fields than do crawling infants (Kretch et al., 2014), and infants' motor costs of social looks are lower when infants are in an upright posture than when they are prone (Franchak et al., 2018). In this study, the increased visibility of parents in high and distant positions may have allowed walking infants to look at their social partners from farther interpersonal distances. Unfortunately, it is difficult to make strong claims about infants' social looking behavior from our results because our data were recorded from infants' second-person perspective, and we have no data about how infants looked at parents when infants were not visible to their parents. However, considering that infants and parents have a “seeing” and “be seen” relationship at the time of eye contact, this study suggests that infants' visual experience of gaze communication is affected by infants' posture or locomotion.

### 4.2. Number of Objects Between Infant and Parent

In Analysis 3, we investigated whether the number of objects between infants and parents is affected by walking development. Contrary to our prediction, regardless of whether eye contact was initiated by the infant or the parent, the number of objects on the floor between the dyad or objects in the infant's hands did not change with the proportion of infant's walking time when controlling for effects of temporal autocorrelation, infant age, and interpersonal distance at which eye contact occurs.

Previous studies suggest that walkers are more likely to carry objects and approach their parents to share objects than crawlers (Clearfield, 2011; Karasik et al., 2011, 2012). However, taking into account interpersonal distance, this study reveals that there is no indication of the space between the dyad becoming more cluttered with objects when walking develops. This may be because infants' natural act of walking is characterized as exploratory rather than destination directed (Cole et al., 2016; Hoch et al., 2019a,b). During free play, short bouts, curved paths, and omnidirectional steps are prevalent in infants' walking (Lee et al., 2018), and infants' walking often does not end near discernible destinations, such as objects or people (Cole et al., 2016; Hoch et al., 2019a,b). Considering that the prevalence of short bouts and the rarity of destinations persist across the development of walking (Cole et al., 2016; Lee et al., 2018), walkers' carrying behavior may not be characterized as destination directed. Such a characteristic of infants' natural walking might make it difficult to detect the effect of walking time on the number of objects on the floor between the dyad or objects in the infants' hands in this study.

Although the proportion of infant's walking time did not have a clear effect on the number of objects between infants and parents, the number of objects on the floor tended to increase with the infant-parent distance regardless of whether eye contact was initiated by the infant or the parent. In daily life, unless objects are concentrated in a particular location in space, it is generally expected that more objects will appear as the interpersonal distance increases. This study does not directly measure the distribution of objects in infants' everyday environment, but this result shows that the number of objects on the floor between the dyad in daily gaze communication is closely related to the positioning of infant and parent. Considering that developmental change in the interpersonal distance at the time of infant-led EC bouts is associated with walking development, these results suggest that as the crawling infant makes the transition to upright locomotion, eye contact initiated by infants occurs in situations that are more distant and with more objects in front of them, which may have profound implication for the development of shared attention.

### 4.3. Implication for Shared Attention

The current study shows that the ecological context in which gaze communication occurs changes with infants' walking development from the point of view of the spatial arrangement of the infant, the parents, and objects. Along with walking development, eye contact from the infants was likely to occur in situations where the infant-parent distance was larger, and therefore, more objects were cluttered on the floor between the dyad. This finding suggests that infants' locomotion or posture dynamically changes the visual environment between the dyads when infants initiate gaze communication.

In this study, we showed that the space at which eye contact was initiated by the infant expanded with infants' walking development, but it may also be related to shared attention in daily face-to-face interaction. Eye contact is an event closely linked to shared attention, which forms a referential triangle of infants, adults, and target objects (Tomasello, 2009). Eye contact encourages the infant's gaze to follow a target object (Senju and Csibra, 2008; Ishikawa and Itakura, 2019), and infants often produce eye contact to initiate joint attention (Mundy et al., 2007). If the expansion of the space at which an infant-led EC bout occurs derives from an embodied factor, such as a change in the infant's first-person visual experience consistent with their motor development, then the visual environment of the shared attention that the infant experiences may also be characterized by a larger interpersonal distance that is more cluttered with objects consistent with the infant's walking development. Such a spatial arrangement of infant, parent, and objects may be associated with shared attention in multiple ways.

First, the arrangement of objects in the infant's first-person perspective may influence the infant's task demand to achieve shared attention. In general, achieving shared attention involves the relative spatial arrangement of the infant, the parent, the target object, and distractors. When we focus on the spatial arrangement of the target object in the infant's first-person perspective, walking development may decrease the infant's task demand for shared attention. By 6 months of age, some infants can follow an adult's head turn toward a target object within the infant's visual field, and, as infants grow, they can follow an adult's gaze to target objects in their periphery and outside of the infants' visual field (Butterworth and Jarrett, 1991; Deák et al., 2000; Flom et al., 2004). If walking development changes the ecological context of shared attention to that similar to a large interpersonal distance so that the parent's face and the target object are simultaneously within the infant's visual field, then the infant might achieve shared attention easily because the parent's gaze can be tracked without the motor cost from tilting the infant's head up or object representation outside of the visual field. On the other hand, when we focus on the spatial arrangement of distractors within an infant's first-person perspective, walking development may increase the infant's task demand for shared attention. Although it depends on the noticeability of the parent's attention-directing gestures (i.e., looking, head turn, and pointing), young infants often fixate on intermediate objects or distractors and fail to engage in shared attention (Butterworth and Jarrett, 1991; Flom et al., 2004). If walking development changes the ecological context of shared attention to that similar to a large interpersonal distance so that many distractors are within the infant's visual field, such a situation might make it difficult to achieve shared attention, especially for young infants.

Second, the arrangement of objects on the floor between the dyad may influence a pathway to achieve shared attention. Recent studies using head-mounted eye trackers worn by infants have reported that there are two pathways to achieve shared attention: the gaze-following pathway and the hand-following pathway (Yu and Smith, 2013, 2017a,b). If the visual environment between the dyad at which the shared attention occurs changes with walking development, then the weight of the pathways the infant uses to achieve shared attention may also change. For example, at a large interpersonal distance, infants may use information from parents' gaze direction rather than parents' hand movement to achieve shared attention because there may be many objects that each person in the dyad cannot manually access, and hand-following would not work for sharing attention about such objects. To test these predictions, it would be necessary to investigate developmental change in the ecological context in which shared attention occurs by using head-mounted eye trackers on both infant and parent.

Acquisition of new motor skills instigates and facilitates cascades of change across a range of domains; this is known as a developmental cascade (for reviews, see Campos et al., 2000; Anderson et al., 2013; Adolph and Hoch, 2019; Franchak, 2020b). A particularly intriguing developmental cascade traces walking experience to language development. The onset of walking is associated with increases in infants' receptive and productive vocabulary (Walle and Campos, 2014). Although the causal mechanism is not fully identified yet, previous studies have focused on social interaction as a factor that links walking to language. Walkers more frequently retrieve (Dosso and Boudreau, 2014), carry, and share distal objects (Clearfield, 2011; Karasik et al., 2011, 2012), and parents provide different verbal responses to walkers compared with crawlers (Karasik et al., 2014). However, although shared attention is closely related to later language development (Brooks and Meltzoff, 2005; Okumura et al., 2017), there is no behavioral study investigating the relation between walking development and shared attention in everyday life (but see Walle, 2016). To reveal the causal mechanism of the developmental cascade set off by walking, it would be necessary to investigate the relation between walking development and shared attention, taking into account the gaze communication space in further study.

### 4.4. Limitations and Conclusions

There are several limitations to this study. One limitation is its small sample size as our data came from only five dyads. There are many previous studies that employ small sample sizes but analyze high-density data in language, motor, and social development (Thelen et al., 1993; Yoshida and Smith, 2008; Franchak et al., 2011; Yu and Smith, 2012; Roy et al., 2015; Clerkin et al., 2017; Suanda et al., 2019). This is especially true in head-mounted eye tracking studies, as the time-intensive, frame-by-frame scoring typically leads to modest sample sizes (Franchak, 2020a), and this study is no exception. Although our dense set of longitudinal recordings provides useful information to understand the developmental process of natural gaze behavior, establishing the generality of our results will require more evidence. Another limitation comes from our method. It is difficult to draw any conclusion about infants' own social looking behavior because our data were recorded from infants' second-person perspective. In addition, the implications for shared attention must be considered as hypotheses to be tested because our results are descriptive and correlational.

In spite of the limitations above, this study shows how the spatial arrangement of the infant, the parent, and objects where gaze communication occurs changes with walking development in everyday life. The transition from crawling to walking changes the ecological context in which infants initiate eye contact to a visual environment characterized by a large infant-parent distance and more objects cluttered between the dyad. Infants' exploration is closely tied with their posture or motor skills, and the exploratory experiences in everyday life are assumed to mediate developmental cascades (Franchak, 2020b). Although many developmental theories have emphasized the role of infants' experience in infant development for a long time, direct measurement of infants' daily experiences is rare (Dahl, 2017). By recording daily face-to-face interaction from infants' second-person perspective, we found longitudinal change in free-moving dyads' gaze communication in everyday life. Further studies describing daily gaze communication from infants' second-person perspective as well as infants' first-person perspective may shed light on how new motor skills provide infants with new opportunities for learning in their lived experiences.

## Data Availability Statement

The datasets and the codes used for the models and graphs are available in the GitHub repository (https://github.com/dororo1225/GazeCommunication2).

## Ethics Statement

The studies involving human participants were reviewed and approved by the ethics review board at the Unit for Advanced Studies of the Human Mind, Kyoto University. Written informed consent to participate in this study was provided by the participants' legal guardian/next of kin. Written informed consent was obtained from the individual(s), and minor(s)' legal guardian/next of kin, for the publication of any potentially identifiable images or data included in this article.

## Author Contributions

HY designed the study, collected field data, carried out the analysis, and drafted the manuscript. AS and SI helped with data collection and drafting the manuscript.

### Conflict of Interest

The authors declare that the research was conducted in the absence of any commercial or financial relationships that could be construed as a potential conflict of interest.
