# Supplementary material for: Transition From Crawling to Walking Changes Gaze Communication Space in Everyday Infant-Parent Interaction
Source: Front Psychol. 2020 Jan 24;10:2987. doi: 10.3389/fpsyg.2019.02987 (PMC7025586; doi:10.3389/fpsyg.2019.02987)
Supplement: Supplementary file 1 [file Data_Sheet_1.pdf]

Supplementary Material

1 STATISTICAL ANALYSIS

We conducted three main statistical analyses using Bayesian state-space models. The core of the state-space model is a generalized linear mixed model (GLMM) (Baayen, 2008) used to estimate the effects of various factors on the response variable. Analysis 1 was intended to estimate the effect of walking development on the infant-parent distance at which eye contact occurs. Analysis 2 was intended to estimate the effect of walking development on the usual infant-parent distance, which is not limited to the gaze communication context. Analysis 3 was intended to estimate the effect of walking development on the number of objects between the dyad.

Regarding the data from infant-parent dyad i on observation day j, each EC session k includes at least an EC bout l. We designed the statistical models to estimate various effects on the response variables. Regarding the statistical models, the set of β_* in the linear predictors represents the coefficients of the explanatory variables. In all analyses, β_1 represents the effect of infant age and β_2 represents the effect of the proportion of infant's walking time. β_3 was incorporated in the statistical model only in Analysis 3, and it represents the effect of interpersonal distance at the time of the EC bout. If the parameter estimate of one explanatory variable is positive, it can be interpreted that the response variable increases with the value of the explanatory variable while controlling for the effects of the other explanatory variables. If the 95% credible interval of the parameters does not include zero, it can be inferred that there is a significant effect, as seen in classic statistical hypothesis testing.

Unlike GLMM, state-space models can be divided into an observation process and state process, and the models consider the effects of temporal autocorrelation based on longitudinal observation data. The observation process links the unobserved state process to the observed data, and the state process describes how latent developmental states change through time. Throughout all statistical models, we also designed the constant term y_{ij}, which represents the intercept of infant-parent dyad i on observation day j, in the linear predictors, such that the effects of temporal autocorrelation were incorporated. The dynamics of y_{ij} are defined as a random variable following the normal distribution of mean $y_{ij-1} + \beta_1$ and standard deviation σ_i, and this stochastic process becomes the core of the state process. In all analyses, we chose a half-t distribution having 4 degrees of freedom as weakly informative priors for the hyperprior of σ_i because they help to stabilize parameter estimates (Gelman et al., 2013). We describe the details of each statistical model in the subsection below.

In all analyses, the models were fitted using the Hamiltonian Monte Carlo engine Stan 2.19.2 (Stan Development Team, 2019) in R 3.6.0 (R Core Team, 2019). All iterations were set to 11000 and burn-in samples were set to 1000, with the number of chains set to four. The values of Rhat for all parameters were below 1.1, indicating convergence across the four chains (Gelman et al., 2013).

1.1 Analysis 1

In Analysis 1, the response variable was infant-parent distance of infant-led EC bout or parent-led EC bout. The statistical model can be described as follows:

$$Y_l \sim LogNormal(\mu_{ij}, \sigma_{obs}) \tag{1.1}$$

$$\mu_{ij} = y_{ij} + \beta_2 * \text{walking}_{ij} + r_k \tag{1.2}$$

$$y_{ij} \sim Normal(y_{ij-1} + \beta_1, \sigma_i) \tag{1.3}$$

$$r_k \sim Normal(0, \sigma_{session}) \tag{1.4}$$

Let us assume that Y_l is the observed interpersonal distance of EC bout l, and it follows a lognormal distribution of the mean μ_{ij}, which is described in Equation 1.1. The linear predictor is described in Equation 1.2, and walking$_{ij}$ represents the walking time proportion of infant i on observation day j. The core of the process model is described in Equation 1.3. To consider differences in the EC session, we set the EC session as random intercept r_k in the linear predictor. We used a normal distribution on mean 0 and standard deviation $\sigma_{session}$ as the prior distribution of r_k, which is described in Equation 1.4.

1.2 Analysis 2

Analysis 2 was a kind of mixed ordered logistic regression controlling for the effects of temporal autocorrelation, and the response variable was the proportion of each distance category for each observation day. The statistical model can be described as follows:

$$Y_{ijk} \sim Binomial(N_{ij}, q_{ijk}) \tag{2.1}$$

$$q_{ijk} = \begin{cases} 1 - \text{logit}^{-1}(\eta_{ij} - c_1) & (k = 1) \\ \text{logit}^{-1}(\eta_{ij} - c_1) - \text{logit}^{-1}(\eta_{ij} - c_2) & (k = 2) \\ \text{logit}^{-1}(\eta_{ij} - c_2) - \text{logit}^{-1}(\eta_{ij} - c_3) & (k = 3) \\ \text{logit}^{-1}(\eta_{ij} - c_3) & (k = 4) \end{cases} \tag{2.2}$$

$$\text{logit}\eta_{ij} = y_{ij} + \beta_2 * \text{walking}_{ij} \tag{2.3}$$

$$y_{ij} \sim Normal(y_{ij-1} + \beta_1, \sigma_i) \tag{2.4}$$

k represents one level of distance category, which is a 4-point ordered scale (1: ”0 - 0.5 m”; 2: ”0.5 - 1.0 m” ; 3: ”1.0 - 1.5 m” ; 4: ”1.5 m or more”). Y_{ijk} is the observed number of instantaneous sampling of one distant category k for infant-parent dyad i on observation day j. Its upper limit is N_{ij}, and it is the total number of instantaneous sampling for infant-parent dyad i on observation day j. Let us assume that Y_{ijk} follows the binomial distribution of parameter q_{ijk}, which is described in Equation 2.1. Equation 2.2 shows how q_{ijk} is determined with parameter η_{ij} and c_k in case of each distant category. c_k is one of a sequence of cutpoints sorted so that $c_1 < c_2 < c_3$. The linear predictor is described in Equation 2.3, and the logit link function is applied for η_{ij}. walking$_{ij}$ represents the same variable as the statistical models of Analysis 1. The core of the process model is described in Equation 2.4. We chose a normal distribution on mean 0 and standard deviation 10 as the prior distribution of c_k. Moreover, we chose a normal distribution on mean 0 and standard deviation 100 as the prior distribution of β_*.

1.3 Analysis 3

In Analysis 3, the response variable was the number of objects on the floor between the dyad or objects in the infant's hands at the time of an infant-led EC bout or parent-led EC bout. The statistical model can be described as follows:

$$Y_l \sim Poisson(\lambda_l) \tag{3.1}$$

$$\log \lambda_l = y_{ij} + \beta_2 * \text{walking}_{ij} + \beta_3 * \text{distance}_l + r_k \tag{3.2}$$

$$y_{ij} \sim Normal(y_{ij-1} + \beta_1, \sigma_i) \tag{3.3}$$

$$r_k \sim Normal(0, \sigma_{session}) \tag{3.4}$$

Let us assume that Y_l is the observed number of objects on the floor between the dyad or objects in the infant's hands at EC bout l, and it follows a Poisson distribution of the mean λ_l, which is described in Equation 3.1. The linear predictor is described in Equation 3.2, and the log link function is applied for λ_l such that the factors in the linear predictor affect λ_l multiplicatively. In Equation 3.2, distance_l represents the interpersonal distance at the EC bout l, and walking_{ij} represents the same variable as in the statistical models of Analysis 1 and Analysis 2. The core of the process model is described in Equation 3.3. To consider differences in the EC session, we set the EC session as random intercept r_k in the linear predictor for only the analysis of objects on the floor because this setting made convergence difficult for the analysis of objects in the infant's hands. We used a normal distribution on mean 0 and standard deviation $\sigma_{session}$ as the prior distribution of r_k, which is described in Equation 3.4.

REFERENCES

Baayen, R. (2008). *Analyzing Linguistic Data: A Practical Introduction to Statistics using R* (Cambridge, UK: Cambridge University Press)

Gelman, A., Carlin, J. B., Rubin, D. B., Vehtari, A., Dunson, D. B., and Stern, H. S. (2013). *Bayesian data analysis* (London: Chapman & Hall/CRC), 3rd edn.

R Core Team (2019). *R: A Language and Environment for Statistical Computing*. R Foundation for Statistical Computing, Vienna, Austria

Stan Development Team (2019). *RStan: the R interface to Stan*. R package version 2.19.2

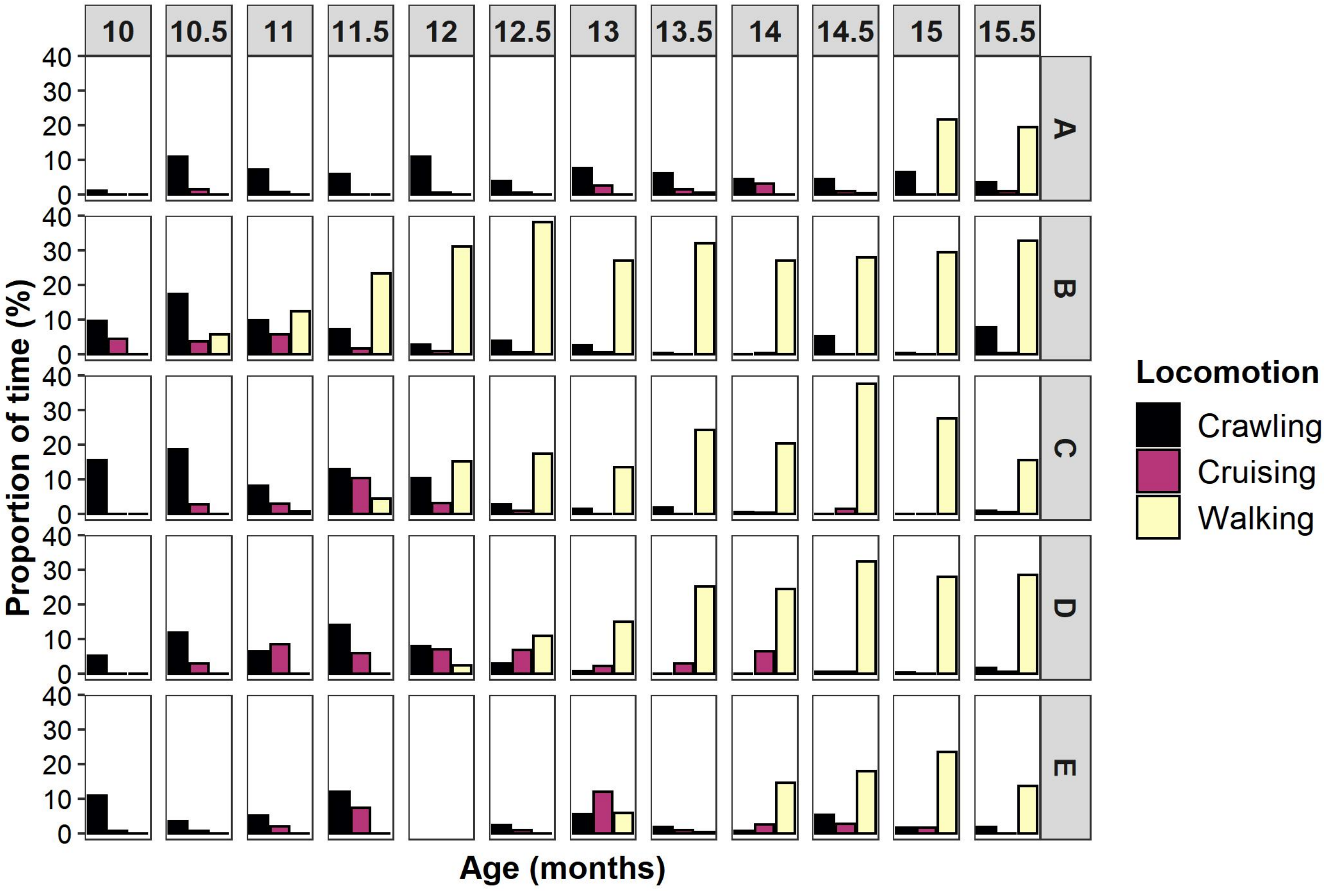

Figure S1. Longitudinal development of the time proportion of infant's locomotion. The color of the bar represents 3 types of infant locomotion: crawling (black), cruising (purple) and walking (white). The subplots on the row dimension represent the longitudinal change of each infant-parent dyad. The subplots on the column dimension represent individual differences at each age in months. Note that there was one observation day (when infant E was 12 months old) on which we could not collect data (missing data).

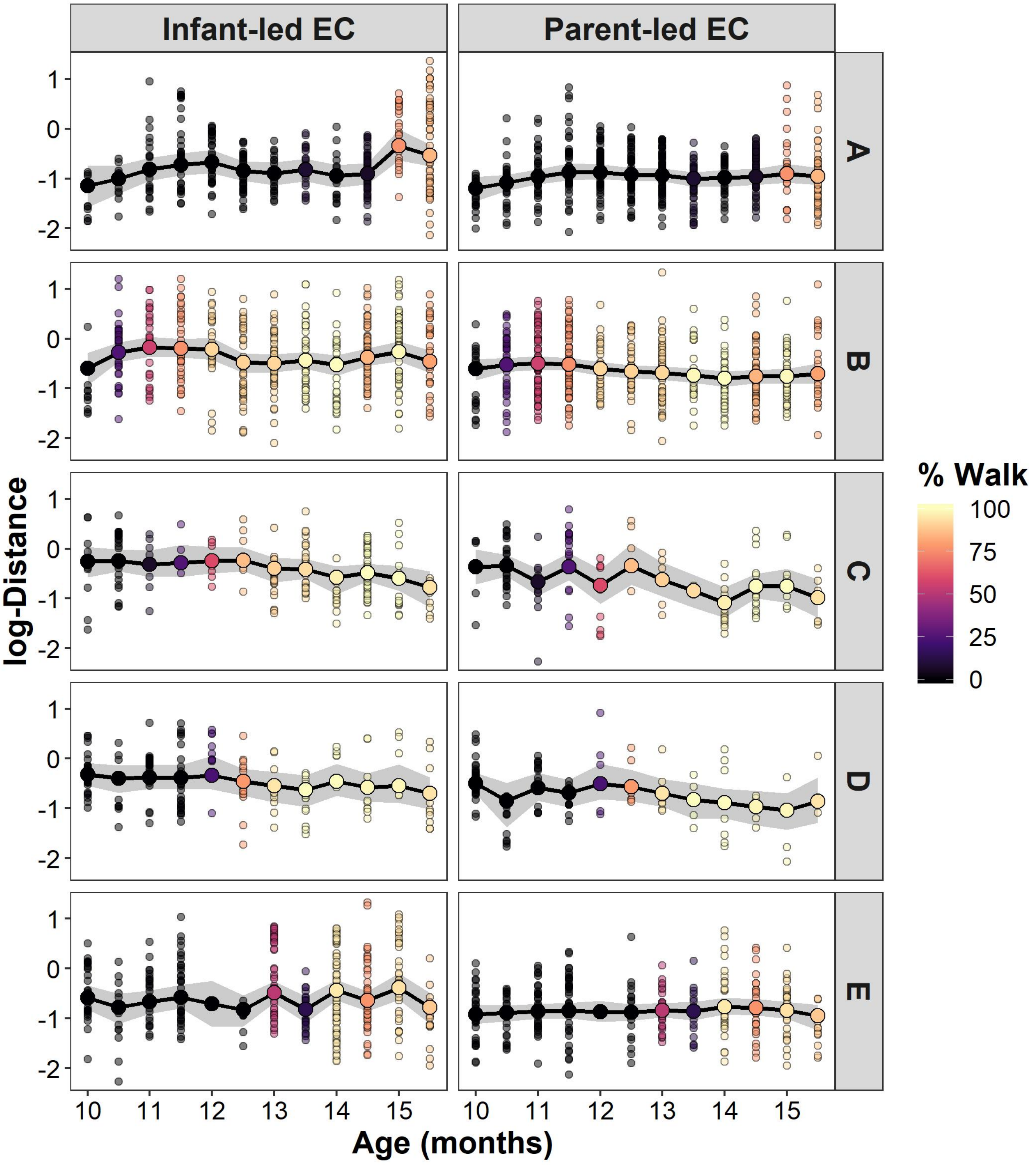

Figure S2. Longitudinal development of the interpersonal distance at which infant-led EC bouts (left panel) and parent-led EC bouts (right panel) occur in Analysis 1. The subplots on the column dimension represent individual differences in developmental change. The posterior mean (large colored dots) and 95% credible interval (gray areas) of the mean interpersonal distance of EC bouts on each observation day are shown. The observed data are represented with small colored dots. The color of the dots represents the proportion of the infant's walking time to the sum of walking time and crawling time for each observation day. Note that the interpersonal distance of each EC bout (meters) is log transformed.

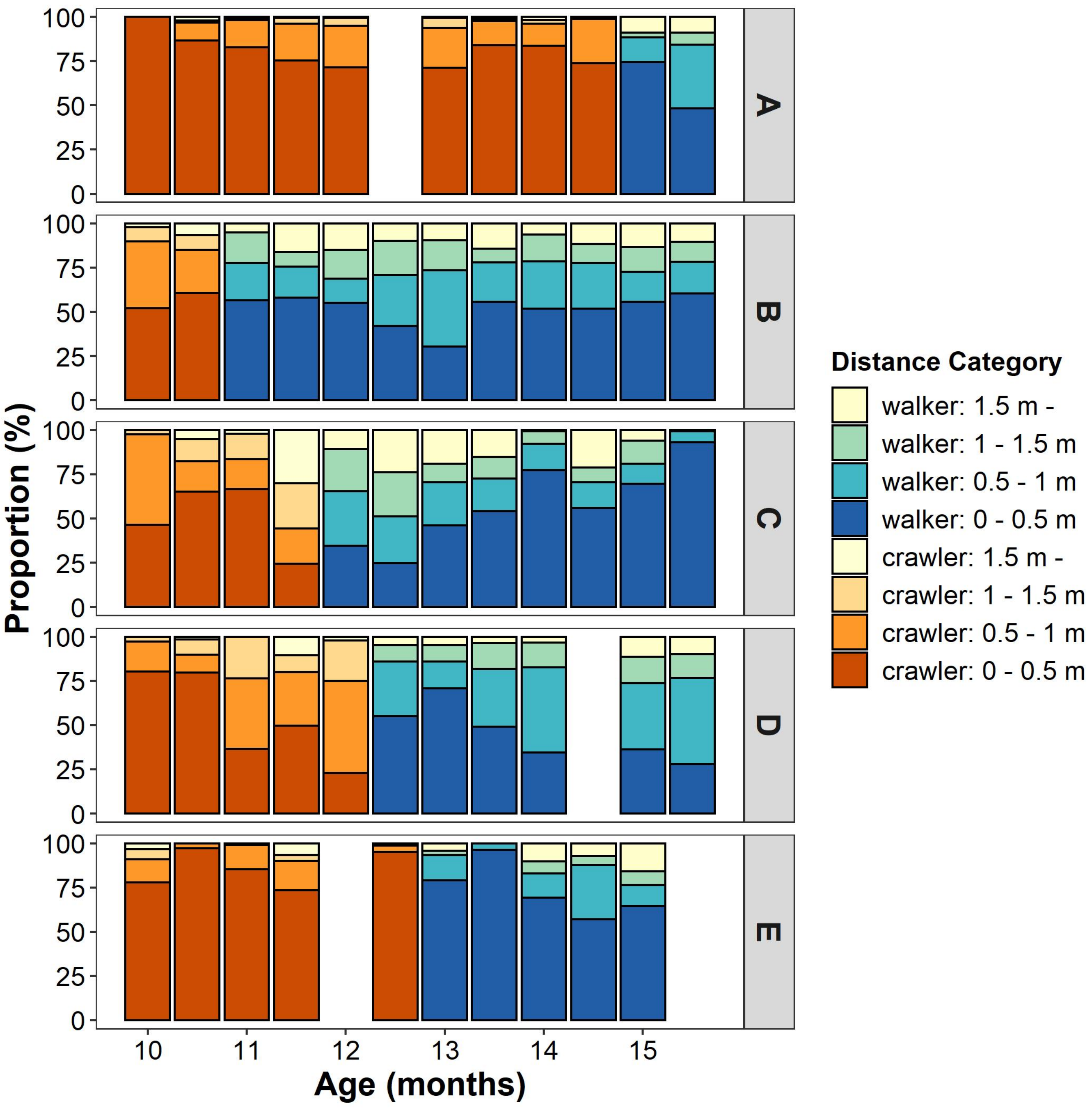

Figure S3. Longitudinal development of the proportion of usual infant-parent distance not limited to the gaze communication context in Analysis 2. The subplots on the column dimension represent individual differences in developmental change. The brightness of the bar (dark - light dimension) represents each distance category, which is an ordered scale for near (dark) to far (light). To visualize longitudinal data simply, we defined locomotion status on each observation day based on the proportion of the infant's walking time. The hue of the bar (red - blue dimension) represents the infant's locomotion status on each observation day. The first observation day on which the percentage of walking time became larger than crawling time was defined as "acquisition of walking"; we categorized the infant's locomotion status before acquisition of walking as "crawler" (red) and otherwise as "walker" (blue). Note that there were some observation days (when infant A was 12.5 months old, when infant D was 14.5 months old and when E was 15.5 months old) on which we could not collect data for Analysis 2 (missing data).

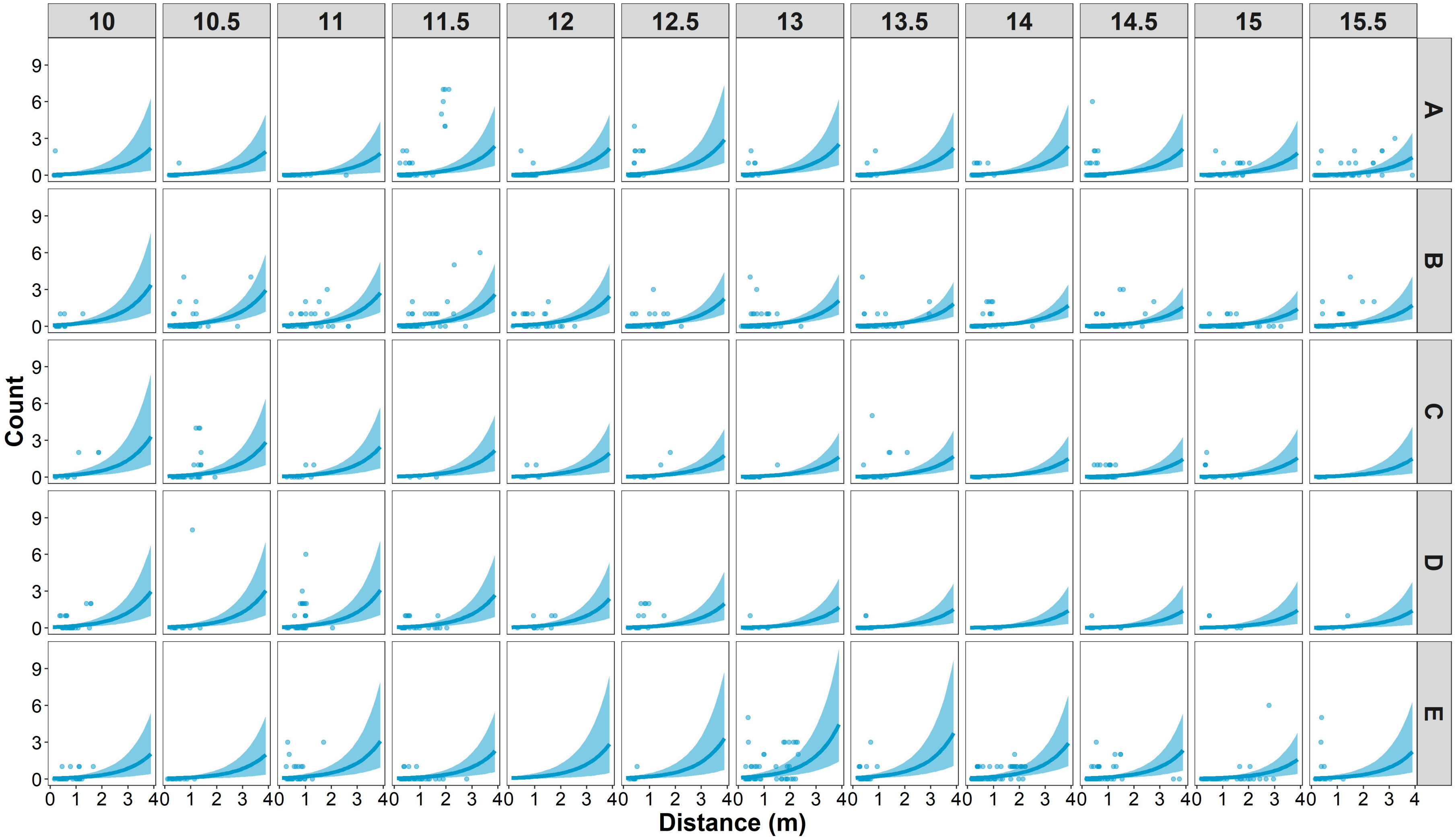

Figure S4. Longitudinal change of the relationship between the infant-parent distance and the number of objects on the floor between the dyad for infant-led EC bouts in Analysis 3. The observed data (coloured dots), posterior mean (coloured lines) and 95% credible interval (coloured areas) of the number of objects for infant-led EC bouts are shown in each subplot. All subplots are for each participant and each observation day. The subplots in the rows represent the longitudinal change of each individual. The subplots in the columns represent the individual differences at each age in months.

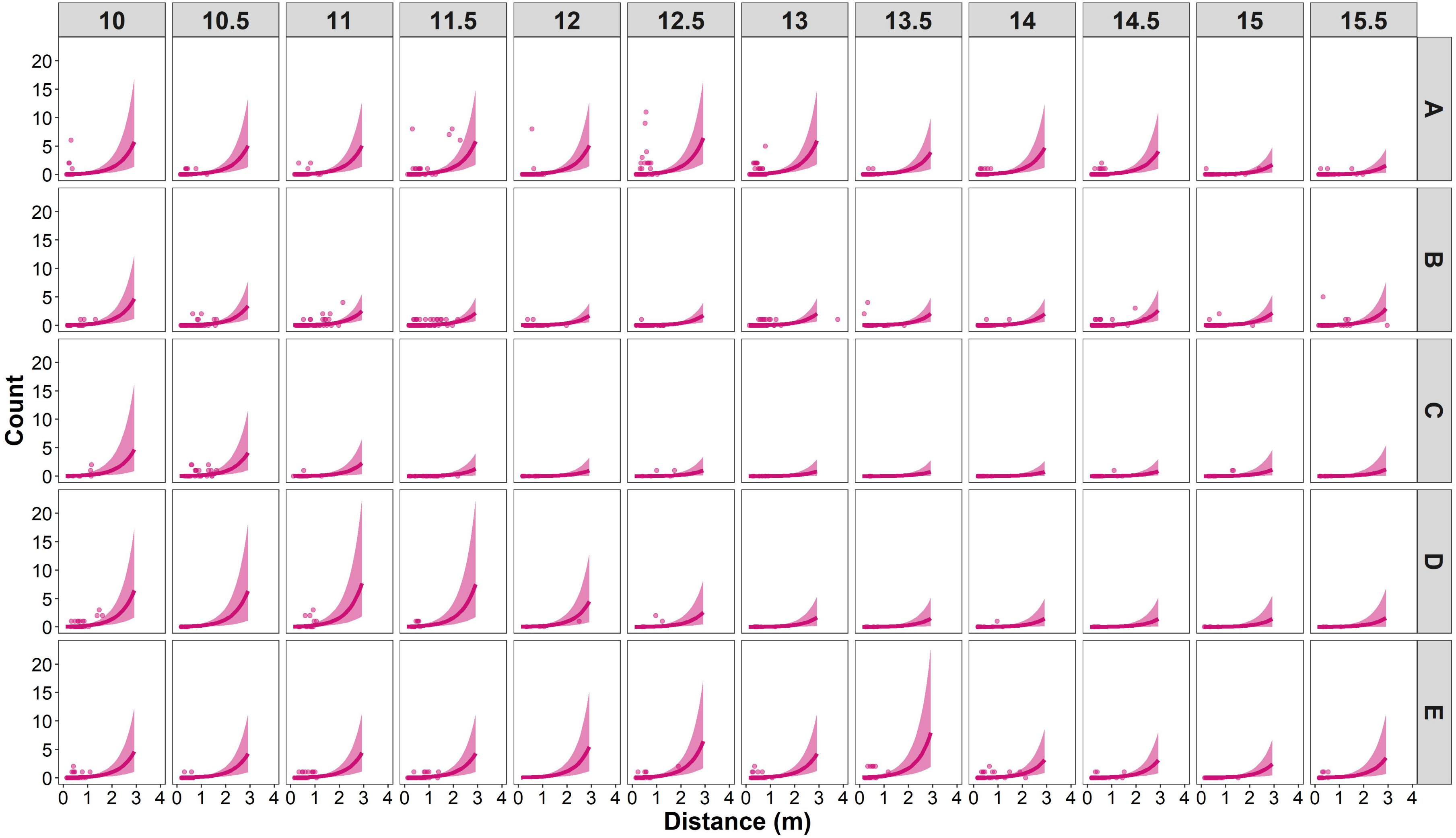

Figure S5. Longitudinal change of the relationship between the infant-parent distance and the number of objects on the floor between the dyad for parent-led EC bouts in Analysis 3. The observed data (coloured dots), posterior mean (coloured lines) and 95% credible interval (coloured areas) of the number of objects for parent-led EC bouts are shown in each subplot. All subplots are for each participant and each observation day. The subplots in the rows represent the longitudinal change of each individual. The subplots in the columns represent the individual differences at each age in months.

Table S1. Descriptive statistics of interpersonal distance at the time of infant-led EC for each observation day. The numbers in the column names represent the age of the infant.

Dyad	Variable	10	10.5	11	11.5	12	12.5	13	13.5	14	14.5	15	15.5
A	Walking Proportion (%)	0	0	0	0	0	0	0	8.3	0	6.3	77	85
	Number of EC	11	12	24	34	35	36	19	20	19	77	24	49
	Mean Distance (m)	0.27	0.36	0.57	0.83	0.61	0.44	0.42	0.51	0.41	0.46	1.1	1.1
	Max Distance (m)	0.42	0.55	2.6	2.1	1.1	0.77	0.79	0.91	1.0	0.87	2.0	3.9
	Min Distance (m)	0.16	0.17	0.19	0.22	0.18	0.20	0.23	0.24	0.16	0.15	0.25	0.12
	SD of Distance (m)	0.10	0.11	0.50	0.63	0.26	0.16	0.16	0.21	0.21	0.16	0.55	0.93
B	Walking Proportion (%)	0	25	56	76	92	91	92	99	100	85	99	81
	Number of EC	14	42	31	39	27	42	48	43	35	67	63	29
	Mean Distance (m)	0.44	0.94	1.1	1.1	1.0	0.74	0.74	0.81	0.63	0.86	1.1	0.94
	Max Distance (m)	1.3	3.3	2.7	3.3	2.5	2.2	2.4	3.0	2.5	2.8	3.2	2.4
	Min Distance (m)	0.22	0.20	0.29	0.23	0.16	0.15	0.12	0.24	0.16	0.25	0.16	0.21
	SD of Distance (m)	0.27	0.58	0.67	0.74	0.61	0.52	0.44	0.63	0.44	0.51	0.66	0.59
C	Walking Proportion (%)	0	0	6.9	25	60	87	90	93	97	100	100	95
	Number of EC	9	26	9	3	7	12	18	22	12	54	13	7
	Mean Distance (m)	0.89	0.98	0.80	1.1	0.82	0.92	0.59	0.82	0.41	0.70	0.62	0.38
	Max Distance (m)	1.9	1.9	1.3	1.6	1.2	1.8	1.5	2.1	0.82	1.3	1.7	0.55
	Min Distance (m)	0.20	0.31	0.28	0.61	0.47	0.42	0.27	0.37	0.22	0.26	0.26	0.25
	SD of Distance (m)	0.63	0.43	0.35	0.51	0.30	0.38	0.29	0.44	0.15	0.29	0.46	0.12
D	Walking Proportion (%)	0	0	0	0	23	78	95	100	100	99	99	95
	Number of EC	29	11	30	25	10	19	10	14	7	9	5	11
	Mean Distance (m)	0.81	0.64	0.84	0.78	1.2	0.65	0.61	0.47	0.91	0.65	1.1	0.56
	Max Distance (m)	1.6	1.4	2.0	2.0	1.8	1.6	1.2	0.74	1.3	1.5	1.7	1.4
	Min Distance (m)	0.37	0.25	0.27	0.28	0.34	0.18	0.25	0.22	0.56	0.30	0.48	0.24
	SD of Distance (m)	0.33	0.36	0.31	0.55	0.44	0.30	0.32	0.17	0.29	0.49	0.57	0.41
E	Walking Proportion (%)	0	0	0	0	-	0	51	14	96	77	94	89
	Number of EC	29	13	26	36	-	10	44	25	69	50	42	17
	Mean Distance (m)	0.71	0.48	0.65	0.80	-	0.39	1.1	0.44	0.91	0.75	1.1	0.42
	Max Distance (m)	1.7	1.1	1.7	2.8	-	0.53	2.3	0.94	2.2	3.8	3.0	1.2
	Min Distance (m)	0.16	0.10	0.25	0.24	-	0.21	0.27	0.24	0.16	0.18	0.17	0.14
	SD of Distance (m)	0.37	0.30	0.34	0.55	-	0.088	0.73	0.17	0.69	0.70	0.78	0.25

Table S2. Descriptive statistics of interpersonal distance at the time of parent-led EC for each observation day.The numbers in the column names represent the age of the infant.

Dyad	Variable	10	10.5	11	11.5	12	12.5	13	13.5	14	14.5	15	15.5
A	Walking Proportion (%)	0	0	0	0	0	0	0	8.3	0	6.3	77	85
	Number of EC	30	30	31	37	61	57	64	52	27	68	22	54
	Mean Distance (m)	0.28	0.37	0.46	0.62	0.50	0.44	0.45	0.35	0.40	0.48	0.63	0.45
	Max Distance (m)	0.41	1.2	1.2	2.3	1.0	1.0	0.81	0.75	0.70	0.82	2.4	2.0
	Min Distance (m)	0.13	0.14	0.15	0.13	0.16	0.17	0.14	0.14	0.16	0.17	0.16	0.14
	SD of Distance (m)	0.067	0.21	0.26	0.50	0.20	0.19	0.18	0.13	0.15	0.16	0.56	0.36
B	Walking Proportion (%)	0	25	56	76	92	91	92	99	100	85	99	81
	Number of EC	17	41	62	66	28	41	53	30	35	48	42	23
	Mean Distance (m)	0.51	0.73	0.74	0.72	0.62	0.62	0.62	0.60	0.46	0.59	0.54	0.75
	Max Distance (m)	1.3	1.6	2.1	2.2	2.0	1.3	3.8	1.8	1.5	2.3	2.1	3.0
	Min Distance (m)	0.18	0.15	0.20	0.18	0.26	0.16	0.13	0.17	0.17	0.19	0.21	0.14
	SD of Distance (m)	0.33	0.38	0.48	0.46	0.38	0.33	0.53	0.37	0.29	0.45	0.34	0.63
C	Walking Proportion (%)	0	0	6.9	25	60	87	90	93	97	100	100	95
	Number of EC	7	26	14	18	9	5	6	6	16	19	8	7
	Mean Distance (m)	0.77	0.84	0.51	1.0	0.46	1.1	0.59	0.43	0.33	0.54	0.65	0.39
	Max Distance (m)	1.2	1.6	1.3	2.2	0.82	1.7	0.92	0.47	0.73	1.4	1.3	0.68
	Min Distance (m)	0.22	0.32	0.10	0.21	0.17	0.42	0.26	0.40	0.18	0.25	0.30	0.22
	SD of Distance (m)	0.38	0.39	0.26	0.50	0.27	0.55	0.26	0.027	0.15	0.34	0.41	0.17
D	Walking Proportion (%)	0	0	0	0	23	78	95	100	100	99	99	95
	Number of EC	31	12	15	11	5	6	10	4	8	5	5	3
	Mean Distance (m)	0.72	0.30	0.70	0.45	1.0	0.67	0.52	0.46	0.53	0.37	0.34	0.62
	Max Distance (m)	1.6	0.47	1.1	0.64	2.5	1.2	1.2	0.72	1.2	0.47	0.69	1.1
	Min Distance (m)	0.31	0.17	0.34	0.29	0.33	0.42	0.35	0.25	0.17	0.25	0.13	0.34
	SD of Distance (m)	0.30	0.10	0.25	0.11	0.88	0.35	0.25	0.22	0.40	0.095	0.22	0.39
E	Walking Proportion (%)	0	0	0	0	-	0	51	14	96	77	94	89
	Number of EC	31	20	32	39	-	22	31	18	29	25	29	11
	Mean Distance (m)	0.45	0.41	0.52	0.57	-	0.49	0.47	0.45	0.62	0.55	0.50	0.31
	Max Distance (m)	1.1	0.66	1.1	1.4	-	1.9	1.1	1.2	2.1	1.5	1.5	0.54
	Min Distance (m)	0.15	0.20	0.15	0.12	-	0.15	0.23	0.20	0.16	0.17	0.14	0.17
	SD of Distance (m)	0.23	0.17	0.24	0.31	-	0.35	0.19	0.23	0.50	0.37	0.28	0.13

Table S3. Descriptive statistics of number of objects between the dyad at the time of infant-led EC for each observation day. The numbers in the column names represent the age of the infant.

Dyad	Variable	10	10.5	11	11.5	12	12.5	13	13.5	14	14.5	15	15.5
A	Walking Proportion (%)	0	0	0	0	0	0	0	8.3	0	6.3	77	85
	Number of EC	11	12	24	34	35	36	19	20	19	77	24	49
	Mean Number of Objects on the Floor	0.18	0.083	0	1.4	0.086	0.44	0.26	0.15	0.26	0.22	0.33	0.35
	SD of Number of Objects on the Floor	0.60	0.29	0	2.4	0.37	0.94	0.56	0.49	0.45	0.80	0.56	0.72
	Mean Number of Objects in Infant's Hands	1.2	0.42	0.71	0.79	0.69	1.1	1.2	1	0.53	0.92	0.71	0.76
	SD of Number of Objects in Infant's Hands	0.75	0.51	0.69	0.69	0.53	0.75	0.76	1.1	0.61	0.82	0.62	0.63
B	Walking Proportion (%)	0	25	56	76	92	91	92	99	100	85	99	81
	Number of EC	14	42	31	39	27	42	48	43	35	67	63	29
	Mean Number of Objects on the Floor	0.21	0.38	0.45	0.62	0.44	0.21	0.33	0.26	0.34	0.19	0.079	0.55
	SD of Number of Objects on the Floor	0.43	0.96	0.77	1.3	0.58	0.56	0.78	0.73	0.68	0.61	0.27	0.95
	Mean Number of Objects in Infant's Hands	0.43	0.74	0.68	0.69	0.70	0.69	0.44	0.51	0.6	0.46	0.62	0.45
	SD of Number of Objects in Infant's Hands	0.51	0.54	0.48	0.61	0.67	0.68	0.58	0.51	0.65	0.59	0.66	0.63
C	Walking Proportion (%)	0	0	6.9	25	60	87	90	93	97	100	100	95
	Number of EC	9	26	9	3	7	12	18	22	12	54	13	7
	Mean Number of Objects on the Floor	0.67	0.69	0.22	0	0.29	0.25	0.056	0.55	0	0.15	0.38	0
	SD of Number of Objects on the Floor	1	1.3	0.44	0	0.49	0.62	0.24	1.2	0	0.36	0.65	0
	Mean Number of Objects in Infant's Hands	0.67	0.38	0.22	0.67	0.71	0.58	0.78	0.23	0.17	0.54	0.15	0.57
	SD of Number of Objects in Infant's Hands	0.5	0.50	0.44	0.58	0.49	0.51	0.43	0.61	0.39	0.50	0.38	0.53
D	Walking Proportion (%)	0	0	0	0	23	78	95	100	100	99	99	95
	Number of EC	29	11	30	25	10	19	10	14	7	9	5	11
	Mean Number of Objects on the Floor	0.38	0.73	0.9	0.24	0.3	0.58	0.1	0.14	0	0.11	0.4	0.091
	SD of Number of Objects on the Floor	0.68	2.4	1.3	0.44	0.48	0.84	0.32	0.36	0	0.33	0.55	0.30
	Mean Number of Objects in Infant's Hands	0.79	0.36	0.5	0.28	0.4	0.26	0.7	0.71	0	0.44	0.6	0.55
	SD of Number of Objects in Infant's Hands	0.68	0.50	0.51	0.46	0.70	0.45	0.67	0.91	0	0.53	0.55	0.69
E	Walking Proportion (%)	0	0	0	0	-	0	51	14	96	77	94	89
	Number of EC	29	13	26	36	-	10	44	25	69	50	42	17
	Mean Number of Objects on the Floor	0.17	0	0.54	0.11	-	0.1	0.93	0.32	0.39	0.34	0.19	0.59
	SD of Number of Objects on the Floor	0.38	0	0.90	0.32	-	0.32	1.3	0.69	0.52	0.69	0.94	1.4
	Mean Number of Objects in Infant's Hands	0.34	0.62	0.54	0.31	-	0.6	0.48	0.52	0.80	0.48	0.57	0.71
	SD of Number of Objects in Infant's Hands	0.55	0.87	0.58	0.52	-	0.70	0.76	0.59	0.68	0.50	0.59	0.47

Table S4. Descriptive statistics of number of objects between the dyad at the time of parent-led EC for each observation day. The numbers in the column names represent the age of the infant.

Dyad	Variable	10	10.5	11	11.5	12	12.5	13	13.5	14	14.5	15	15.5
A	Walking Proportion (%)	0	0	0	0	0	0	0	8.3	0	6.3	77	85
	Number of EC	30	30	31	37	61	57	64	52	27	68	22	54
	Mean Number of Objects on the Floor	0.37	0.17	0.16	1.0	0.15	0.74	0.38	0.038	0.22	0.12	0.091	0.056
	SD of Number of Objects on the Floor	1.2	0.38	0.52	2.3	1.0	2.0	0.86	0.19	0.42	0.37	0.29	0.23
	Mean Number of Objects in Infant's Hands	1.4	0.7	0.52	0.97	0.69	1.2	1.3	0.69	0.41	0.82	0.64	0.98
	SD of Number of Objects in Infant's Hands	0.81	0.60	0.68	0.69	0.50	0.76	0.77	0.88	0.64	0.67	0.73	0.76
B	Walking Proportion (%)	0	25	56	76	92	91	92	99	100	85	99	81
	Number of EC	17	41	62	66	28	41	53	30	35	48	42	23
	Mean Number of Objects on the Floor	0.18	0.20	0.24	0.23	0.071	0.024	0.19	0.2	0.057	0.23	0.071	0.30
	SD of Number of Objects on the Floor	0.39	0.51	0.67	0.42	0.26	0.16	0.39	0.81	0.24	0.56	0.34	1.1
	Mean Number of Objects in Infant's Hands	0.41	0.63	0.69	0.48	0.54	0.59	0.53	0.77	0.51	0.42	0.60	0.43
	SD of Number of Objects in Infant's Hands	0.51	0.54	0.50	0.56	0.51	0.63	0.67	0.50	0.70	0.54	0.59	0.59
C	Walking Proportion (%)	0	0	6.9	25	60	87	90	93	97	100	100	95
	Number of EC	7	26	14	18	9	5	6	6	16	19	8	7
	Mean Number of Objects on the Floor	0.43	0.5	0.071	0	0	0.4	0	0	0	0.053	0.25	0
	SD of Number of Objects on the Floor	0.79	0.71	0.27	0	0	0.55	0	0	0	0.23	0.46	0
	Mean Number of Objects in Infant's Hands	0.43	0.58	0.21	0.56	0.44	0.2	0.5	0.33	0.12	0.58	0.25	0
	SD of Number of Objects in Infant's Hands	0.53	0.58	0.43	0.51	0.53	0.45	0.55	0.52	0.34	0.61	0.46	0
D	Walking Proportion (%)	0	0	0	0	23	78	95	100	100	99	99	95
	Number of EC	31	12	15	11	5	6	10	4	8	5	5	3
	Mean Number of Objects on the Floor	0.45	0	0.6	0.36	0.2	0.5	0	0	0.12	0	0	0
	SD of Number of Objects on the Floor	0.77	0	0.99	0.50	0.45	0.84	0	0	0.35	0	0	0
	Mean Number of Objects in Infant's Hands	0.61	0.25	0.67	0.27	0.2	0.17	0.4	0.5	0.38	0.4	0.8	0.67
	SD of Number of Objects in Infant's Hands	0.62	0.45	0.49	0.47	0.45	0.41	0.52	0.58	0.52	0.55	0.84	0.5
E	Walking Proportion (%)	0	0	0	0	-	0	51	14	96	77	94	89
	Number of EC	31	20	32	39	-	22	31	18	29	25	29	11
	Mean Number of Objects on the Floor	0.26	0.1	0.28	0.15	-	0.27	0.19	0.61	0.34	0.12	0	0.27
	SD of Number of Objects on the Floor	0.51	0.31	0.46	0.37	-	0.55	0.48	0.92	0.55	0.33	0	0.47
	Mean Number of Objects in Infant's Hands	0.35	0.9	0.72	0.56	-	1	0.90	0.61	0.76	0.56	0.62	0.45
	SD of Number of Objects in Infant's Hands	0.49	0.64	0.63	0.64	-	0.69	0.70	0.70	0.58	0.58	0.68	0.52
